# Effect of physical activity on changes in weight and aerobic capacity during an 18-month behavioral weight loss intervention

**DOI:** 10.1186/s12966-025-01754-3

**Published:** 2025-05-21

**Authors:** Seth A. Creasy, Danielle M. Ostendorf, Laura Kaizer, Rebecca Rosenberg, Matthew J. Breit, Daniel H. Bessesen, Edward L. Melanson, Victoria A. Catenacci

**Affiliations:** 1 Department of Medicine, Division of Endocrinology, Metabolism, and Diabetes, University of Colorado Anschutz Medical Campus, 12348 E. Montview Blvd., Aurora, CO 80045 USA; 2 Anschutz Health and Wellness Center, University of Colorado Anschutz Medical Campus, Aurora, CO USA; 3 Department of Biostatistics and Informatics, Colorado School of Public Health, University of Colorado Anschutz Medical Campus, Aurora, CO USA; 4 Division of Endocrinology, Denver Health Medical Center, Denver, CO USA; 5 Division of Geriatrics, University of Colorado Anschutz Medical Campus, Aurora, CO USA

**Keywords:** Exercise, Lifestyle treatment, Obesity, Timing

## Abstract

**Background:**

The objective of this secondary analysis was to examine the effect of MVPA and the time of day that MVPA (i.e., morning vs. evening) is performed on long-term changes in body weight and aerobic capacity.

**Methods:**

Adults with overweight and obesity (*n* = 105) enrolled in an 18-month behavioral weight loss intervention involving a reduced calorie diet and a supervised exercise program. Participants were encouraged to increase their bouted MVPA (i.e., MVPA accumulated in bouts ≥ 10 min) to 300 min/wk. Body weight, body composition, aerobic capacity, and physical activity were assessed at 0, 6, 12, and 18 months. Participants were categorized based on whether they increased bouted MVPA by ≥ 150 min/wk during the supervised exercise program. Linear regression was used to examine the effect of increasing bouted MVPA on weight loss and aerobic capacity. Similar methods were used to examine the effect of time of day of MVPA on weight loss and aerobic capacity.

**Results:**

Participants who increased bouted MVPA by ≥ 150 min/wk during the supervised exercise program had greater weight loss, fat loss, and increases in aerobic capacity at 18 months compared to participants who increased bouted MVPA by < 150 min/wk. Amongst participants who increased bouted MVPA by ≥ 150 min/wk, the time of day of MVPA had no significant effect on weight loss or aerobic capacity.

**Conclusions:**

Increasing bouted MVPA by ≥ 150 min/wk improves weight loss and aerobic capacity during a behavioral weight loss intervention which includes caloric restriction. MVPA earlier and later in the day is beneficial for weight management and cardiorespiratory fitness.

**Trial registration:**

Clinicaltrials.gov (NCT01985568) on October 24, 2013.

## Background

Obesity prevalence continues to climb in the US despite significant public health efforts [1]. Lifestyle treatment, including dietary modification, increased moderate to vigorous physical activity (MVPA), and behavioral support remains the cornerstone for obesity treatment and typically elicits 5–10% weight loss [2]. A variety of individual-level, social, environmental, and other factors may influence the magnitude of weight loss during behavioral weight loss treatment [3]. In addition to these factors, behavioral adherence is an important factor in weight management. Specifically, engagement in high amounts of bouted MVPA (i.e., MVPA accumulated in bouts ≥ 10 min) has been associated with long-term weight loss maintenance [4–9].

Evidence from the National Weight Control Registry (NWCR) has shown that individuals maintaining a significant amount of weight loss (e.g., ≥ 13.6 kg for ≥ 1 year) engage in ~ 275 min/wk of objectively measured bouted MVPA [8]. The importance of bouted MVPA for long-term weight loss has also been confirmed in secondary analyses of behavioral weight loss interventions with individuals achieving the highest weight loss also engaging in the highest amount of bouted MVPA [4–6]. A limitation of this prior literature is that most studies examine the association between concurrent MVPA engagement and weight loss maintenance. Studies examining whether engagement in MVPA early on in an intervention is predictive of future weight loss or weight loss maintenance are lacking.

In addition to the volume of MVPA, the timing (i.e., time of day) of bouted MVPA may influence weight loss and weight loss maintenance. Some evidence suggests that morning exercise leads to greater weight loss compared to evening exercise [10, 11]. Further, Creasy et al. found that weight loss maintainers engage in high amounts of MVPA within 3 h of waking up, suggesting a potential body weight regulation benefit for this time of day [9]. However, not all studies agree that MVPA at a specific time of day elicits superior weight management benefits [12–14]. Evidence on whether the timing of MVPA affects changes in body weight, body composition, or aerobic capacity during a behavioral weight loss intervention is needed.

The primary purpose of this secondary data analysis was to examine the association between changes in bouted MVPA early on in a behavioral weight loss intervention on long-term changes in weight loss, fat mass loss, and aerobic capacity (i.e. 6–12 months later). In addition, we examined whether the time of day of bouted MVPA, (i.e., regular engagement in the morning vs. evening) affected changes in weight, fat mass, and aerobic capacity. We hypothesized that engaging in higher amounts of MVPA and morning MVPA would be associated with greater reductions in weight and fat mass and greater increases in aerobic capacity.

## Methods

### Participants

This was a secondary analysis of adults (18–55 years) with overweight or obesity (BMI 27–42 kg/m^2^) who completed an 18-month behavioral weight loss intervention (NCT01985568) in the Denver metro area from 2014 to 2018. Details on the methods, participants, inclusion/exclusion criteria, and primary findings have been published previously [15]. The intervention included a calorie-restricted diet (1200–1800 kcal/d), provision of supervised aerobic exercise for 6 months, a recommendation to engage in bouted MVPA (300 min/wk), and group behavioral support. The study was originally designed to examine the effect of initiating exercise at the onset of the behavioral weight loss intervention (Standard) vs. initiating exercise 6 months later following initial weight loss from diet-only (Sequential) on long-term weight loss and aerobic capacity. All aspects of this study were approved by the Colorado Multiple Institutional Review Board and participants provided written informed consent prior to engaging in study-related procedures. Individuals who passed the telephone questionnaire were invited to complete a screening visit, including a health history and physical exam by the study physician. Individuals were excluded for known cardiovascular disease, metabolic disease, some forms of cancer, previous weight loss surgery, > 5% change in body weight during the previous 6 months, regular engagement in > 150 min/wk of exercise, current use of medications known to affect appetite, weight, or energy metabolism, or any medical conditions that contraindicated exercise.

### Behavioral weight loss intervention

Participants were randomized in a 1:1 ratio to the Standard or Sequential intervention arms. Both groups received an identical 18-month group-based behavioral weight loss program that included a 6-month supervised exercise program. The only difference between groups was the timing of initiation of the supervised exercise program: Standard initiated supervised exercise at month 0, whereas Sequential initiated supervised exercise at month 6. Frequency of aerobic exercise progressed from 3 to 5 days per week and all exercise was prescribed at a moderate intensity which was monitored using heart rate monitors (Polar Electro, Bethpage, NY). Exercise session duration progressed from 20 to 60 min (60 min/wk to 300 min/wk). Following the exercise ramp up, participants were recommended to complete supervised exercise 5 days per week and 60 min per day (300 min/wk) at moderate intensity (65–75% heart rate maximum). Participants could complete supervised exercise between 6:00 AM and 7:00 PM. Following the 6-month supervised exercise program, participants were encouraged to continue engaging in ≥ 300 min/wk of bouted MVPA on their own through month 18. No recommendations about exercising before or after meals (i.e., fasted vs. fed exercise) were made. Participants received complimentary access to the fitness facility from the beginning of the supervised exercise program through the remainder of the study. Participants were prescribed a calorie-restricted diet (1200–1800 kcal/d) throughout the 18-month study which was individually determined based on estimated basal metabolic rate [16]. Participants also received group-based behavioral support (provided by a trained registered dietitian). Behavioral support was provided weekly for weeks 0–20, every other week for weeks 21–26, and monthly for weeks 27–78. Additional details about the behavioral weight loss intervention have been described previously [15].

### Assessments

All assessments were conducted at baseline, 6, 12, and 18 months.

#### Weight and body composition

Body weight was assessed on a digital scale calibrated annually, with participants wearing a light gown. Body composition (i.e., fat mass and fat free mass) was assessed using dual-energy x-ray absorptiometry (DXA; Hologic QDR Series; Bedford, MA).

#### Patterns of physical activity

Patterns of physical activity, including sedentary behavior (SB, < 1.5 metabolic equivalents), light-intensity physical activity (LPA, 1.5–2.99 metabolic equivalents), and MVPA (≥ 3.0 metabolic equivalents) were assessed using the SenseWear armband (BodyMedia Inc; Pittsburgh, PA). Participants were asked to wear the armband for 24 h/d over seven consecutive days at each assessment. Physical activity data were included in the analysis if they met validity criteria (> 22.8 h/d of weartime and > 4 days of wear with ≥ 1 weekend day). MVPA was calculated in ≥ 1 min, ≥ 5 min, and ≥ 10 min bouts. Because MVPA was encouraged in bouts of ≥ 10 min and due to minimal changes in the other bout categories (i.e., MVPA in bouts of < 10 min), we chose to categorize participants based on changes in bouts of ≥ 10 min. Participants were classified based on measured change in MVPA accumulated in bouts of ≥ 10 min from baseline to the end of the 6-month supervised program (month 6 if randomized to Standard or 12 if randomized to Sequential). These timepoints were selected to correspond with the supervised exercise program when adherence to the bouted MVPA recommendation was likely the highest. Participants were categorized based on whether they increased MVPA accumulated in bouts ≥ 10 min by ≥ 150 min/wk from baseline to the end of the 6-month supervised exercise program (yes/no). When examining the time-of-day effects, only participants classified as increasing bouted MVPA by ≥ 150 min/wk were included. Participants were classified as AM-Active if the majority (≥ 50%) of their bouted MVPA minutes throughout the week occurred from prior to 12:00 PM, and participants were classified as PM-Active if the majority of their bouted MVPA minutes occurred after 12:00 PM based on data from the SenseWear armband. This method accurately defined those who were active in the morning vs. evening. The four most active one-hour time windows for the AM group were 5 AM, 6AM, 7AM, and 8AM. The four most active one-hour time windows for the PM group were noon, 4PM, 5PM, 6PM. Other timing categories were considered; however, due to sample size, we could only dichotomize the variable. In addition, we ran sensitivity analyses to examine the whether using different AM and PM timing criteria affected our results, and we found no evidence that choosing different criteria significantly affected our results.

#### Maximal aerobic capacity

Maximal aerobic capacity was assessed using a treadmill protocol and indirect calorimetry. A modified Balke protocol was utilized. Participants were instructed to continue exercising until volitional exhaustion. The following criteria were used to assess whether true maximum was achieved: increase in VO_2_ of < 2 ml/kg/min with an increase in workload, peak heart rate ≤ 10 beats per minute from age-predicted maximum heart rate, and respiratory exchange ratio of ≥ 1.10. Two out of three criteria needed to be met for the test to be considered valid, and thus, included in the analysis.

### Statistical analysis

Statistical analyses were performed using SAS 9.4 (SAS System for Microsoft; SAS Institute Inc., Cary, NC). Changes in patterns of physical activity between those with ≥ 150 min/wk and < 150 min/wk, as well as between those AM-Active and PM-Active, were described using random intercept mixed effects models with unstructured covariance matrices. Estimated means and estimated mean change from baseline, as well as their corresponding 95% confidence intervals, are included. The confidence intervals for change from baseline excluding zero can be interpreted as significant. Data in tables are presented as mean ± standard deviation (SD) unless otherwise noted. Data in figures are presented as mean ± standard error of the measurement (SEM). Linear regression was used to examine potential differences for change in weight, fat mass, aerobic capacity, and physical activity behavior between ≥ 150 min/wk and < 150 min/wk. Similarly, linear regression was utilized to examine potential differences for change in weight, fat mass, aerobic capacity, and patterns of physical activity between participants classified as AM-Active and PM-Active. For the purposes of these analyses, all participants with complete data were included. We previously found that the intervention arm did not differentially affect diet, physical activity, weight loss, or cardiorespiratory fitness [15]; however, all models were run unadjusted and adjusted for randomized group. Results were similar so only those adjusted for randomized group are reported. A p-value of < 0.05 was considered statistically significant for all analyses. An a-priori power calculation was not performed for this specific analysis.

## Results

### Baseline characteristics

A total of 170 adults with overweight and obesity were randomized to Sequential or Standard. This secondary analysis included 105 participants who had valid weight change data (month 18 - baseline) and valid device-measured physical activity data at baseline and the end of the 6-month supervised exercise program. Baseline demographics of included participants are shown in Table 1. Participants were then grouped into ≥ 150 min/wk and < 150 min/wk based on measured changes in bouted MVPA at the end of the 6-month supervised exercise program, as described in the methods.

**Table 1:** Demographics and baseline characteristics (*n* = 105)

Characteristic	Mean ± SD or *n* (%)
Age (years)	40.5 ± 9.0
Weight (kg)	96.0 ± 15.8
BMI (kg/m^2^)	34.5 ± 4.4
Intervention	
Sequential	50 (47.6)
Standard	55 (52.4)
Sex	
Female	86 (81.9)
Male	19 (18.1)
Race	
White	80 (76.2)
Black	16 (15.2)
Other	9 (8.6)
Ethnicity	
Hispanic or Latino	27 (25.7)
Not Hispanic or Latino	78 (74.3)

### Effect of bouted MVPA on weight loss and aerobic capacity

**Fig. 1:**
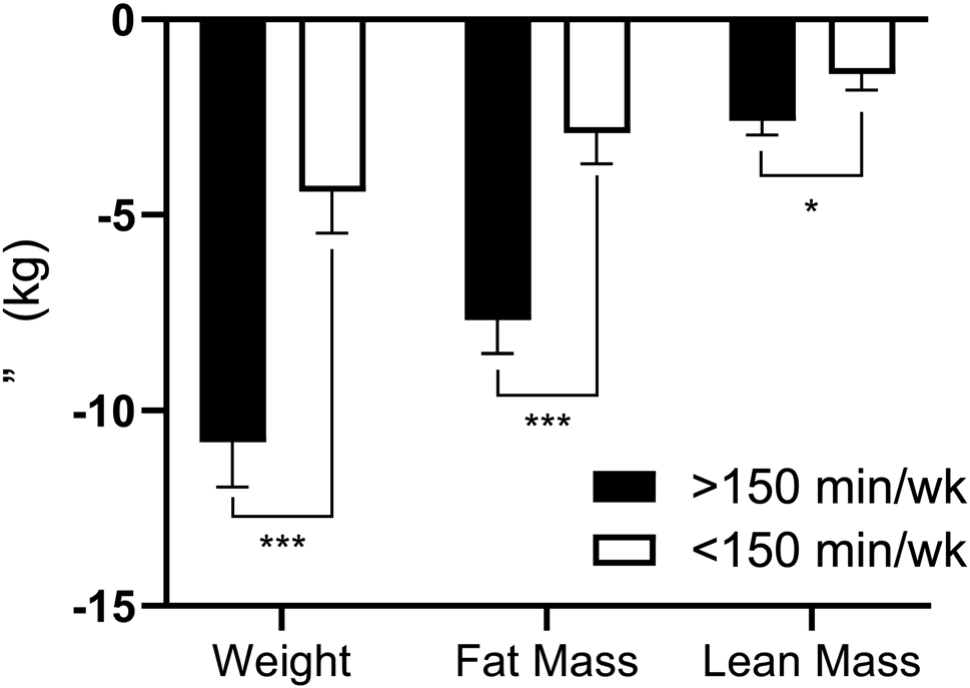
Changes in Weight and Body Composition (< 150 min/wk vs. ≥150 min/wk). **Legend** Error bars indicate SEM. *significant difference between < 150 min/wk and ≥ 150 min/wk at *p* < 0.05. ***significant difference between < 150 min/wk and ≥ 150 min/wk at *p* < 0.001

Changes in patterns of physical activity are shown in Table 2. Baseline patterns of physical activity (sedentary behavior, LPA, MVPA, and bouted MVPA) were similar between groups. By definition, participants classified as ≥ 150 min/wk increased total MVPA and bouted MVPA significantly more than participants classified as < 150 min/wk at the end of the 6-month supervised exercise program. At 18 months, participants classified as ≥ 150 min/wk at the end of the supervised exercise program continued to have significantly greater total MVPA and bouted MVPA compared to participants classified as < 150 min/wk. Participants classified as ≥ 150 min/wk exhibited significant decreases in sedentary behavior and minimal changes in LPA during the supervised exercise program. These changes in patterns of physical activity were similar at month 18. Participants classified as < 150 min/wk exhibited no significant changes in sedentary behavior; however, increases in LPA that were observed following the supervised exercise program were not sustained at 18 months. Results of the linear regression analysis are shown in Table 3. Participants classified as ≥ 150 min/wk demonstrated a 6.4 kg (95% CI: -9.5, -3.3; *p* < 0.001) greater decrease in weight and a 4.8 kg (95% CI: -7.1, -2.5; *p* < 0.001) greater decrease in fat mass at 18 months compared to participants with < 150 min/wk of bouted MVPA (Fig. 1). Participants classified as ≥ 150 min/wk had a 1.1 kg (95%CI: -2.2, -0.1; *p* = 0.037) greater decrease in lean mass at 18 months than participants with < 150 min/wk of bouted MVPA. In addition, participants classified as ≥ 150 min/wk demonstrated a 3.0 mL/kg/min (95% CI: 1.5, 4.5; *p* < 0.001) greater increase in aerobic capacity at 18 months compared to participants with < 150 min/wk of bouted MVPA. There were no differences in absolute aerobic capacity (L/min) between ≥ 150 min/wk and < 150 min/wk.

**Table 2:** Change in patterns of physical activity (< 150 min/wk vs. ≥150 min/wk)

Physical Activity Variable	Timepoint	< 150 min/wk (*n* = 56)		≥ 150 min/wk (*n* = 49)		
		Estimated Mean (95% CI)	Change from Baseline (95% CI)	Estimated Mean (95% CI)	Change from Baseline (95% CI)	Difference between groups (95% CI)
SED (min/d)	0 M	722.3 (697.6, 746.9)		734.5 (708.2, 760.9)		12.3 (-23.8, 48.4)
	6 M or 12 M	708.1 (683.4, 732.8)	-14.2 (-31.9, 3.6)	683.0 (656.6, 709.4)	-51.6 (-70.5, -32.6)	-25.1 (-61.2, 11.0)
	18 M	723.7 (698.6, 748.8)	1.4 (-17.0, 19.7)	707.2 (680.2, 734.1)	-27.4 (-47.1, -7.7)	-16.5 (-53.3, 20.3)
LPA (min/d)	0 M	200.9 (184.2, 217.7)		198.8 (180.8, 216.7)		-2.2 (-26.7, 22.4)
	6 M or 12 M	219.3 (202.5, 236.1)	18.4 (4.3, 32.5)	205.0 (187.0, 222.9)	6.2 (-8.8, 21.3)	-14.3 (-38.9, 10.2)
	18 M	209.8 (192.7, 227.0)	8.9 (-5.6, 23.5)	200.7 (182.3, 219.2)	2.0 (-13.6, 17.6)	-9.1 (-34.3, 16.1)
MVPA (min/d)	0 M	60.8 (50.2, 71.5)		64.6 (53.2, 75.9)		3.7 (-11.9, 19.3)
	6 M or 12 M	67.1 (56.5, 77.8)	6.3 (-2.1, 14.6)	118.2 (106.8, 129.6)	53.6 (44.7, 62.5)	51.0 (35.5, 66.6)
	18 M	58.9 (48.1, 69.8)	-1.9 (-10.5, 6.8)	93.4 (81.7, 105.0)	28.8 (19.5, 38.1)	34.4 (18.5, 50.4)
Bouted MVPA (min/d)	0 M	17.0 (10.7, 23.3)		17.1 (10.4, 23.8)		0.1 (-9.1, 9.2)
	6 M or 12 M	21.7 (15.4, 28.0)	4.7 (-1.0, 10.4)	66.2 (59.5, 72.9)	49.2 (43.1, 55.2)	44.5 (35.3, 53.7)
	18 M	14.6 (8.2, 21.1)	-2.4 (-8.2, 3.5)	40.7 (33.8, 47.6)	23.6 (17.3, 29.9)	26.0 (16.6, 35.5)

SED = sedentary behavior, < 1.5 METs; LPA = light-intensity physical activity, 1.5–2.99 METs; MVPA = moderate-to-vigorous physical activity, ≥ 3.0 METs; Bouted MVPA = MVPA accumulated in bouts of ≥ 10 min

**Table 3:** ≥150 min/wk vs. <150 min/wk linear regression estimates

Dependent	Adjusted Parameter Estimate (95% CI)	*p*-value
Weight (kg)	-6.4 (-9.5, -3.3)	< 0.001
Fat Mass (kg)	-4.8 (-7.1, -2.5)	< 0.001
Lean Mass (kg)	-1.1 (-2.2, -0.1)	0.04
VO2 Max (ml/kg/min)	3.0 (1.5, 4.5)	< 0.001
VO2 Max (L/min)	0.0 (-0.1, 0.1)	0.37

### Effect of timing of bouted MVPA on weight loss and aerobic capacity

**Fig. 2:**
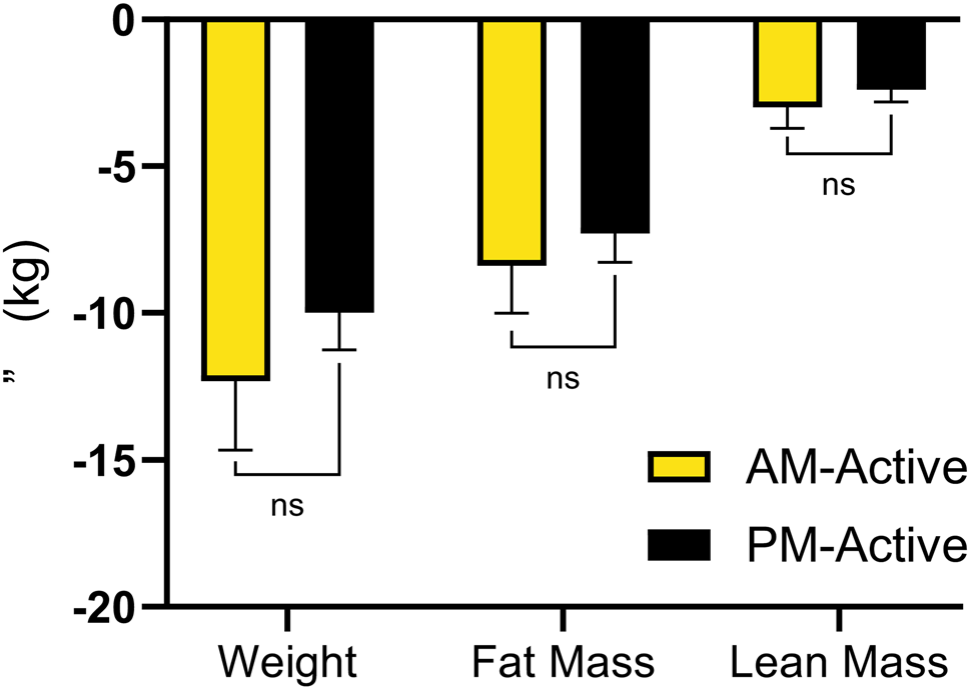
Changes in Weight and Body Composition (AM-Active vs. PM-Active). ns– not significant (*p* > 0.05). **Legend** Error bars indicate SEM

Following the above analyses, participants classified as ≥ 150 min/wk were categorized into AM-Active or PM-Active based on the time of day which they completed the majority of their bouted MVPA. Changes in patterns of physical activity between AM-Active and PM-Active are shown in Table 4. Both groups significantly decreased sedentary behavior during the supervised exercise program; however, only AM-Active sustained these reductions at month 18. AM-Active also demonstrated increases in LPA following the supervised exercise program and at month 18. Results of the linear regression analysis are shown in Table 5. There were no significant differences for change in weight, fat mass, or lean mass between AM-Active and PM-Active at 18 months (Fig. 2). There were no significant differences for change in relative or absolute aerobic capacity between AM-Active and PM-Active at 18 months.

**Table 4:** Change in patterns of physical activity (PM-Active vs. AM-Active)

Physical Activity Variable	Timepoint	PM-Active *n* = 32		AM-Active *n* = 17		
		Estimated Mean (95% CI)	Change from Baseline (95% CI)	Estimated Mean (95% CI)	Change from Baseline (95% CI)	Difference between groups (95% CI)
SED (min/d)	0 M	746.0 (714.6, 777.4)		713.0 (669.9, 756.0)		-33.1 (-86.4, 20.3)
	6 M or 12 M	694.4 (663.0, 725.8)	-51.7 (-70.7, -32.6)	661.6 (618.5, 704.7)	-51.40 (-77.5, -25.2)	-32.7 (-86.1, 20.6)
	18 M	732.8 (700.8, 764.7)	-13.2 (-33.3, 6.8)	660.6 (617.2, 704.0)	-52.40 (-79.1, -25.7)	-72.2 (-126.1, -18.3)
LPA (min/d)	0 M	196.4 (175.4, 217.4)		203.2 (174.4, 232.1)		6.9 (-28.9, 42.6)
	6 M or 12 M	190.3 (169.3, 211.3)	-6.1 (-22.0, 9.8)	232.6 (203.8, 261.5)	29.40 (7.6, 51.2)	42.3 (6.6, 78.0)
	18 M	185.8 (164.2, 207.4)	-10.6 (-27.3, 6.1)	228.4 (199.2, 257.6)	25.10 (2.8, 47.4)	42.6 (6.3, 78.9)
MVPA (min/d)	0 M	62.1 (45.4, 78.9)		69.1 (46.2, 92.1)		7.0 (-21.4, 35.4)
	6 M or 12 M	115.0 (98.2, 131.7)	52.8 (41.0, 64.6)	124.2 (101.2, 147.2)	55.10 (38.9, 71.2)	9.2 (-19.2, 37.7)
	18 M	85.2 (68.1, 102.4)	23.1 (10.7, 35.4)	107.9 (84.7, 131.1)	38.70 (22.2, 55.3)	22.7 (-6.2, 51.5)
Bouted MVPA (min/d)	0 M	16.6 (5.8, 27.5)		17.9 (3.1, 32.7)		1.3 (-17.1, 19.6)
	6 M or 12 M	67.7 (56.9, 78.6)	51.1 (41.6, 60.6)	63.4 (48.6, 78.3)	45.50 (32.5, 58.6)	-4.3 (-22.7, 14.0)
	18 M	38.2 (27.0, 49.4)	21.6 (11.6, 31.6)	44.9 (29.8, 60.0)	27.00 (13.6, 40.3)	6.7 (-12.1, 25.4)

SED = sedentary behavior, < 1.5 METs; LPA = light-intensity physical activity, 1.5–2.99 METs; MVPA = moderate-to-vigorous physical activity, ≥ 3.0 METs; Bouted MVPA = MVPA accumulated in bouts of ≥ 10 min

**Table 5:** AM-Active vs. PM-Active linear regression estimates

Dependent	Adjusted Parameter Estimate 95% CI	*p*-value
Weight (kg)	-2.2 (-7.2, 2.8)	0.38
Fat Mass (kg)	-1.0 (-4.6, 2.7)	0.59
Lean Mass (kg)	-0.6 (-2.2, 0.9)	0.42
VO2 Max (ml/kg/min)	1.7 (-0.6, 4.1)	0.14
VO2 Max (L/min)	0.1 (-0.0, 0.3)	0.11

## Discussion

This secondary analysis examined the effect of change in bouted MVPA on changes in body weight, body composition, and aerobic capacity during an 18-month behavioral weight loss intervention. We found that individuals who exhibited an increase of ≥ 150 min/wk of bouted MVPA during the 6-month supervised exercise program demonstrated greater reductions in weight and fat mass at 18 months compared to individuals with lesser increases in bouted MVPA. Additionally, individuals classified as ≥ 150 min/wk demonstrated greater increases in relative aerobic capacity at month 18. Further, amongst participants classified as ≥ 150 min/wk, the time of day of bouted MVPA (morning vs. afternoon/evening) did not significantly affect weight loss, body composition, or changes in aerobic capacity. These findings suggest that engaging in greater amounts of bouted MVPA during a behavioral weight loss intervention that includes both caloric restriction and supervised exercise, results in enhanced long-term weight loss and fitness benefits. In addition, these findings suggest that engaging in bouted MVPA at any time of the day positively impacts weight loss and aerobic capacity during a behavioral weight loss intervention.

MVPA is one of strongest predictors of weight loss maintenance. Jakicic et al. first showed that long-term weight loss and weight loss maintenance were associated with high amounts (~ 200 min/wk) of self-reported MVPA [5, 17]. More recently, Jakicic et al. found that individuals maintaining a weight loss of ≥ 10% at month 18 of a behavioral weight loss program engaged in the highest amount of MVPA (200–300 min/wk) accumulated in bouts of ≥ 10 min [6]. Consistent with those results, we found that greater increases in bouted MVPA at the end of the 6-month supervised exercise program was associated with greater weight loss and fat mass loss at month 18. This finding suggests that increasing structured bouts of MVPA (i.e., bouts ≥ 10 min), which are more commonly thought of as exercise, may be important for long-term weight loss. In this study, every 30 min/wk increase in bouted MVPA was associated with an increase in weight loss of 3.5 kg. The importance of bouted MVPA is also supported by a previous study that found that individuals achieving a ≥ 10% weight loss at 18 months had significantly greater steps accumulated in bouts of ≥ 10 min (~ 3,500 steps/d) compared to individuals who lost less weight over the course of the study [18]. Further supporting the weight loss benefits of longer bouts of MVPA, Creasy et al. recently found that successful weight loss maintainers (i.e., individuals maintaining a weight loss of ≥ 13.6 kg for ≥ 1 year) engaged in longer bouts (≥ 10, ≥ 30, ≥60 min bouts) of MVPA compared to controls with obesity [9]. The 2018 Physical Activity Guidelines for Americans have shifted to focus on total MVPA accumulation rather than bouted MVPA accumulation [19]. A recent cross-sectional analysis found that the manner in which MVPA is accumulated (i.e., bouted vs. non-bouted) may alter cardiometabolic profiles [20]. For weight management specifically, more research is needed to understand the behavioral and physiological differences between bouted and total MVPA accumulation.

Similar to the weight loss findings, we found that individuals with greater increases in bouted MVPA demonstrated greater increases in aerobic capacity compared to individuals with lesser increases in bouted MVPA. When examining changes in absolute aerobic capacity (L/Min), the effect was diminished suggesting the change in weight was the primary driver of the increase in relative fitness. Standard behavioral weight loss interventions which include physical activity typically result in modest increases in aerobic capacity [21–23], similar to what was observed in the current study. While the focus of lifestyle treatment of obesity is often on weight loss, the cardiometabolic protection from greater aerobic fitness cannot be understated [24–27]. Higher aerobic capacity greatly attenuates the mortality risk associated with having a higher BMI [28]. Due to the protective effects of increased fitness, lifestyle treatment of obesity should have a continued focus on strategies to increase MVPA and improve aerobic capacity. Further, future research is needed to identify the effective strategies for increasing and maintaining fitness after weight loss.

The timing of MVPA and exercise may affect weight loss and other clinical outcomes like aerobic capacity, fat mass, and glucose control. Some studies have found that morning exercise produces superior weight loss benefits [10, 11] while other studies have found that evening exercise elicits greater fat mass loss [29, 30]. Other studies have found no difference in weight change between morning versus evening exercise [12–14]. The current results aligns with the latter, as individuals who increased bout MVPA and were more active in the morning had similar changes in weight and body composition at 18 months compared to individuals who were more active in the afternoon/evening. This is a positive message as increasing MVPA can be encouraged at any time of day for weight management. There are limitations to our analysis which may explain why we failed to observe significant differences in weight change between morning activity and afternoon/evening activity including limited statistical power, use of a dichotomous time-based variable rather than a circadian based definition of morning and afternoon/evening, lack of divergence in AM vs. PM activity stimuli between groups (i.e., contamination between groups), or lack of consistency in MVPA timing throughout the study as individuals were only classified based on MVPA timing during the supervised exercise program. Further, time of day for exercise was not specified as part of the prescription in this study, thus, other confounders, such as chronotype, may have impacted our results.

Two recent studies by Schumacher et al. found that early morning physical activity is most prevalent in successful weight loss maintainers and that consistent timing of physical activity is associated with higher amounts of MVPA engagement among successful weight loss maintainers [31, 32]. In addition, consistent timing of activity was associated with greater automaticity (i.e., greater likelihood of habit formation) [31]. Both early morning activity and greater automaticity were associated with a more stable exercise pattern over time [32]. Thus, consistent timing of activity may be particularly important in a standard behavioral weight loss intervention when previously inactive individuals are attempting to increase MVPA. Further, consistent timing of MVPA may have beneficial metabolic effects due to underlying circadian physiology. A review by Chtourou and Souissi found that consistent training at a certain time of day improved exercise performance at that time of day [33]. This suggests that consistent timing of MVPA day-to-day may prime the body to perform best at that time of day. To date, there have been limited investigations into the importance of consistent timing of MVPA. Future prospective studies are needed to understand how consistent timing of activity influences weight loss and other clinical outcomes during a behavioral weight loss intervention.

This analysis does not come without limitations. This was a secondary data analysis, and the original study was not designed or powered for the present analysis. Another limitation is that the provision of supervised exercise occurring at different timepoints in the study (0–6 months in Standard and 6–12 months in Sequential). We controlled for this limitation by classifying participants based on bouted MVPA at the timepoint engagement in bouted MVPA would likely be highest (i.e., near the end of supervised exercise program) and we statistically controlled for randomized arm; however, there may have been other confounders that influenced our results. We also recognize that there are many other ways we could have categorized participants based on MVPA engagement. The chosen categories did identify two distinct groups (i.e., individuals who are highly active in the morning vs. individuals who are highly active in the afternoon/evening). In sensitivity analyses using different time characterization, results were similar. A larger sample is necessary to examine more discrete time windows. Using minutes per week to define MVPA engagement can result in differences in exercise energy expenditure. For example, 150 min of exercise may result up to 2000 kcal of exercise energy expenditure in larger, male subjects while 150 min of exercise may only result in ~ 800 kcal of exercise energy expenditure in smaller female subjects. These differences in exercise energy expenditure may confound our findings. Thus, it is possible that using other MVPA criteria to define these activity categories would have led to different findings. The current criteria were chosen to maximize sample size in each group while also having significant heterogeneity between groups. In addition, these criteria were chosen based on previous guidelines and recommendations for physical activity. In addition, it is possible that the measurement of MVPA used to categorize subjects was not reflective of habitual behavior as the armband was only worn for one week at each timepoint.

Another limitation of this study is that our outcomes (body weight and cardiorespiratory fitness) could have been influenced by other factors besides MVPA engagement. For example, the behavioral intervention included recommendations for caloric restriction, and dietary adherence likely influenced weight change. Moreover, there may have been an interaction between physical activity behavior and energy intake adherence [34–38]. However, our measures of dietary behavior were limited and had significant underreporting [15]. In addition, energy status prior to exercise (i.e., fasted vs. fed exercise) may have influenced our results, but energy status was not collected as part of this study. Similarly, changes in fitness could have been due to a learning effect, as the majority of participants had not completed a maximal aerobic capacity test prior to enrollment in this study. We are unable to discern whether changes in fitness were due to a learning effect vs. a training effect. Despite these limitations, this analysis provides further support that increasing bouted MVPA by ≥ 150 min/wk from baseline levels improves weight loss and clinical outcomes.

## Conclusions

In summary, we found that individuals who demonstrated greater increases in MVPA accumulated in bouts of ≥ 10 min during the 6-month supervised exercise program lost more weight, more fat mass, and had greater improvements in aerobic capacity compared to individuals who engaged in lower amounts of bouted MVPA during an 18-month behavioral weight loss program. Amongst the highly active participants, engaging in more morning or afternoon/evening activity did not significantly affect change in weight, body composition, or fitness. Pending future evidence from randomized studies, MVPA should continue to be encouraged at any time of the day.

## Data Availability

All data used and analyzed during the current study are not publicly available due to institutional IRB guidelines at the time of consent but are available upon written request to Dr. Victoria Catenacci (vicki.catenacci@cuanschutz.edu).

## Abbreviations

NWCR: National Weight Control Registry
BMI: Body Mass Index
kg: kilograms
m: Meters
DXA: Dual-Energy X-ray Absorptiometry
SB: Sedentary Behavior
LPA: Light Physical Activity
MVPA: Moderate-to-Vigorous Physical Activity
VO_2_: Maximal Aerobic Capacity
SD: Standard Deviation
SEM: Standard Error of the Measurement
